# The Principle of Heavy Clothing

**Published:** 1886-05

**Authors:** Alfred S. Gubb

**Affiliations:** Resident Medical Officer, French Hospital, London


### THE PRINCIPLES OF HEALTHY CLOTHING.

BY ALFRED S. GUBB, L.R.C.P., M.R.C.S.

Resident Medical Officer, French Hospital, London.

The choice of materials for clothing has been dependent hitherto far more on the facilities which they offer for manufacture, or self-decoration, than on any preconceived idea of their suitability, although here, as elsewhere, we find on examination that the consensus of opinion of succeeding generations generally leads in the right direction. The best method, therefore, of arriving at the truth is to reason backwards, and to endeavor to ascertain why certain materials have invariably been chosen, under certain circumstances, as clothing material, and this may enable us to understand the laws which underlie this apparent hazard in the matter of choice. The value of clothing consists, as we all know, in promoting the maintenance of our bodies at an equable temperature and in shielding the surface from the effect of sudden and violent changes in the temperature of the surrounding atmosphere. Now the value of any particular substance as an article of clothing is in inverse proportion to its capacity as a conductor of heat. But this is not the only quality to be considered in the selection of such materials, for while we wish to protect our bodies from undue loss of heat by radiation, we have to provide or allow for ventilation and the escape of watery vapor and gases, and here the materials vary within wide limits. It should be born in mind that radiation takes place, not only in proportion to the relatively low temperature of the ambient air, but also, and more markedly, towards bodies which are possessed of a lower temperature than ourselves.

A great factor in protecting our bodies from thermometric changes lies in the different strata of air which intervene between the different layers or garments and permeate the fabrics themselves.

It is to this that we owe the extreme warmth of such substances as eider-down, or furs, or plumage of any sort, the interstices of each being filled with air, which is, in itself, a bad conductor of heat. Many experiments have been made since those first conducted by Rumford, in 1736, to determine the delays which occurred in the cooling of a thermometer heated to a certain degree, when enveloped in hemp, flax, cotton, silk and wool respectively. Since this observer, Senebier and Bœckmann have both recorded numerous experiments with a similar object in view. Black flannel was found to allow of the absorption and dispersion of heat more rapidly than lighter shades, but it is difficult to say how much of this is attributable to the difference in the texture from the chemical changes involved in the dyeing. In any case, the differences found to exist between textures of various hues are only appreciable as regards solar heat, obscure heat not exhibiting the same preferences.

In some more recent investigations by Pettenkofer on a cylinder of sheet iron filled with hot water and enveloped successively in different textures, the variations only representod some one or two per cent., and this irrespective of color, but when the cylinder was exposed to the rays of the sun, the absorption of heat varied between linen and silk as much as 90 to 108. The effect of color was even more striking, and was as follows; White, 100; yellow 140; green, 160; red, 168; blue (light), 198; black, 208. The fig, ures readily explain why a black garment is unsuitable to wear in the sun. It is worthy of note that on anveloping the cylinders with a double layer of satin, cotton, or fine linen, the loss was only diminished by some two to six per cent., whereas, with chamois leather, flannel, or woollen cloths, the diminution amounted to from twenty to thirty per cent. The inference is evident, viz., that the resistance to the passage of heat depends less upon the individual conductivity of the textile fibres than upon the thickness, the volume and the texture of the materials. The conductivity of wadding, for instance, increases some forty per cent. when strongly compressed. Although the radiation which goes on is not notably diminished by doubling the thickness of the investing texture, if it be tightly drawn round the cylinder, it is quite otherwise if an air space is allowed to intervene between the two layers. With such an interspace of only one-third of an inch the radiation is diminished (exclusive of the diminution due to the layers themselves) to the extent of some thirty-five per cent. This, however, is based on the assumption that the intervening column of air is motionless, but as loose garments tend to favor the circulation of the air, they are, notwithstanding this fact, preferred in hot climates. The essential point, indeed, is that the greatest obstacle to the dissemination of heat is found in a discontinuity or want of homogeneity in its elements. Heat, like sound, is but a form of molecular vibrations, and is governed by the same laws. Then, again, the permeability of the various textures to air is a point of capital importance, and this varies from one in glazed kid, to one hundred in flannel. It is essential that our garments should allow of such ventilation, but it is also necessary that this should not take place so rapidly as to affect the sensory elements of the skin, hence the more slowly it takes place, the more conducive is it to our health and comfort.

Another important property of textile fabrics is their capacity of absorbing and retaining moisture; the nearer the air is to its saturation point, the more moisture do they absorb, owing to diminished evaporation. This is accentuated when the temperature, from any cause, falls. The water contained in textures may be divided into two categories, one part not appreciable to the touch, and which cannot be squeezed out; the hygrometric water, properly so called, and a second part which blocks the pores, and which may be expelled by compression, the interposed water. According to the experiments of Mr. Coulvier, wool is more hygroscopic than hemp, and linen than cotton. The quantity of water that these textures can absorb is much greater than is generally supposed. A woollen garment weighing ten to twelve pounds will take up about a quart of water, to evaporate which completely will withdraw from the body from 500 to 600 units of heat. More moisture is absorbed at a low temperature than when it approaches 70 degs. Fahr., further, since wet garments are better conductors of heat than dry ones, they protect us far less efficiently against the cold, hence the danger to health of damp and cold combined.

